# Iron Deficiency Anemia in Southern Jordanian Adolescent Girls: A Study of Prevalence and Contributing Factors

**DOI:** 10.1155/anem/2484033

**Published:** 2025-12-19

**Authors:** Abdullah M. Khamaiseh, Anas A. Khamayseh

**Affiliations:** ^1^ Department of Community and Mental Health Nursing, Faculty of Nursing, Mutah University, Mutah, Karak, Jordan, mutah.edu.jo; ^2^ Department of Orthopedic Surgery, Jordanian Royal Medical Service, Amman, Jordan, jrms.mil.jo

**Keywords:** adolescents, associated factors, iron deficiency anemia, Jordan, prevalence, school students

## Abstract

Anemia is a global public health concern, affecting approximately one‐quarter (24.8%) of the global population, with adolescent girls and women being particularly vulnerable to iron deficiency anemia (IDA) and malnutrition. This study aimed to assess the prevalence of IDA among schoolgirls aged 10–17 in Southern Jordan and identify the associated factors. In this cross‐sectional school‐based study, participants completed a questionnaire that collected demographic data, while laboratory tests were conducted to measure hematological parameters. The results revealed an overall prevalence of IDA at 21.5%, encompassing both mild and moderate cases. A significant association between IDA and age was observed (*p∼0.001*), with the highest prevalence of both moderate and mild anemia found among 16‐year‐olds. These findings underscore the importance of implementing school‐based health promotion programs that focus on improving dietary habits and regular screening to address and prevent IDA).

## 1. Introduction

Anemia is a worldwide public health issue that affects both developed and developing nations [1–3]. It describes a state in which the body’s physiological demands are not met by the quantity of red blood cells or their ability to deliver oxygen. When hemoglobin (Hb) is less than 12 g/dL or the red blood cell count is less than 4.2 million/μL, anemia is diagnosed [2].

The most prevalent cause of anemia, accounting for half of the cases, is iron deficiency anemia (IDA) [4], which is the result of insufficient iron being available to support the normal formation of red blood cells [5]. Poor dietary intake is one of the reasons of IDA [2]. Since our diet is the primary source of iron, poor dietary intake, blood loss that results in iron loss, issues with iron absorption, and other medical disorders like inflammation and end‐stage renal failure are the usual causes of IDA [6].

About 1.3 billion of the world’s population are adolescents, defined as those aged 10–19 years. Adolescents experience long‐term exposure to health risks and their consequences, yet they often have the least power to influence their environment or make decisions that affect their well‐being [7].

The worldwide prevalence of anemia among adolescents is 27% in developing countries and 6% in developed countries [2]. Adolescent girls and women are frequently affected by IDA and malnutrition [8], which can have serious negative effects on their personal health. Adolescent girls are particularly susceptible to iron deficiency because of their rapidly increasing iron needs, inadequate dietary intake, menstrual losses, infections, early marriage customs, and adolescent pregnancy [9]. Teenage girls also require 10% more iron because of blood loss during their periods [10].

Anemia in teenagers is defined as Hb < 11.5 g/dL according to the WHO classification system. Thus, cases of anemia are further classified as severe anemia, defined as less than 7 g/dL; moderate anemia, defined as Hb ranging between 7 and 10 g/dL; and mild anemia, defined as Hb > 10 g/dL. When serum ferritin levels are less than 12 μg/L, anemia is considered as iron deficiency. Both low serum ferritin and Hb levels are used to diagnose subjects with IDA [11].

Anemia affects roughly one‐quarter (24.8%) of the world’s population [10]. The major nutritional issue is expected to be IDA [2]. Teenage anemia has long‐term health and financial repercussions and is a major cause of morbidity and mortality in low‐ and middle‐income nations [12]. A number of studies revealed that teenage malnutrition is a significant public health issue in developing nations [13–15] because teenage girls are a neglected demographic. Enhancing their nutritional status contributes to ending the intergenerational cycle of malnutrition [16, 17]. Teenage anemia results in a decrease in both physical and mental ability, as well as a decrease in focus when working and studying, and additionally presents a significant danger to girls’ ability to become safe mothers in the future [1]. Nutrition and micronutrient deficiencies such as IDA continue to be among the leading cause of years lost in death and disability among adolescents [18]. There is little evidence on the burden of anemia among adolescents in low‐income countries as Jordan [5]. Therefore, this study aimed to assess the prevalence of IDA among adolescent schoolgirls in Southern Jordan and to identify the factors associated with the condition.

## 2. Materials and Methods

### 2.1. Design and Settings

A school‐based cross‐sectional study was conducted from 1 February 2024 to 7 April 2024 among school‐going female adolescents in public secondary schools in Al‐Karak in Southern Jordan. Al‐Karak is 140 km southwest of Amman, the capital of Jordan, and one of the country’s governorates. Al‐Karak, the governorate’s capital, is home to about 320,000 people. The four directorates of education in Al‐Karak—the Directorate of Education for Al‐Karak Region, the Directorate of Education for the Southern Mazar, the Directorate of Education for Al‐Qasr District, and the Directorate of Education for Southern Ghour—are represented in the study area by a sample of secondary schools. Every directorate had two randomly chosen schools, as long as they are comprehensive and have at least 500 female students enrolled.

### 2.2. Study Participants and Sampling Method

Adolescent girls in public secondary schools aged between 10 and 17 years who are in good health and agreed to have their blood tested were included in the study. Exclusion criteria included students without parental consent, schools that receive nutrition supplements on a regular basis, and female students experiencing heavy or clotted menstrual cycles. In addition, students who refuse to have their blood tested, have a history of thalassemia in any form, or are undergoing treatment for anemia were not allowed to enroll. Multistage random sampling was used in the selection of the requisite number of girls from each selected school. In the first stage, all schools in the four directorates having grades from class 4th to 12th were included. In second stage, two government schools were selected randomly from each directorate. Simple random sampling was used to select 284 adolescent girls. The sample size was calculated based on the following formula:

*n*= (Zα/2)2 × *p* (1‐p) d2. A confidence interval of 95% was considered, the level of significance was taken at *α* = 0.05, and margin of error 5%. = (1.96)2 × 0.16(1–0.16) (0.05)^2^

*n* = 257.5311–258. To account for a potential 10% nonresponse rate or attrition, 10% of 258 = 25.8 was added.

Zα/2 = Standard normal variable at 95% confidence level (1.96)

*p* = Population proportion prevalence, *d* = Margin of error, *n* = Sample size.

Ten percent = Non‐respondent rate.

Therefore, the adjusted total sample size is 258 + 25.8 = 283.8, which was rounded up to 284 participants.

### 2.3. Study Instrument

Data were collected by using a structured questionnaire prepared by the researcher, which encompasses four sections.

1. Sociodemographic characteristics: Data including age, class, level of education of parents, and monthly income.
2. Anthropometric data: Anthropometric measurements (height and weight) taken according to WHO standard.
3. Dietary information: Macronutrient and micronutrient intake over 24 h.
4. Menstrual history and history of past illnesses.

### 2.4. Instrument Validity

To ensure the validity of the data collection form, its content was reviewed by a panel of five experts in Nursing and Public Health. Their feedback guided revisions to improve the clarity, relevance, and comprehensiveness of the items. A pilot test was conducted with 30 students who were not part of the main study to assess the clarity and practicality of the form. Minor modifications were made based on their suggestions to enhance understanding and ease of completion. It is important to note that data obtained from the pilot test were not included in the main study. Since the instrument was a structured data gathering form rather than a psychometric questionnaire, reliability indices such as Cronbach’s alpha or test–retest reliability were not deemed necessary.

### 2.5. Assessment of RBC Parameters

Blood samples were collected from each participant under aseptic conditions by trained laboratory personnel. Approximately 3 mL of venous blood was drawn into two tubes: one containing ethylenediaminetetraacetic acid (EDTA) for complete blood count (CBC) analysis, and another plain tube for serum ferritin measurement.

The Hb concentration and mean corpuscular volume (MCV) were determined using an automated hematology analyzer. The analyzer automatically calculated the red blood cell indices based on impedance and photometric methods. Calibration and quality control were performed daily according to the manufacturer’s guidelines to ensure accuracy and precision.

The serum ferritin level was measured from the separated serum using an enzyme‐linked immunosorbent assay (ELISA) method with a commercially available kit. All procedures were conducted following the kit manufacturer’s protocol, and the absorbance was read using a microplate reader at 450 nm.

Based on WHO guidelines, biochemical tests conducted on samples with low Hb and MCV can also be used as a measure to diagnose anemia. At the same time, normal MCV is between 80 and 100 fL. Students with Hb levels lower than cut‐off values are considered to be anemic if Hb < 12.0 g/dL, MCV < 76 fL.

### 2.6. Procedure

Data collection procedure began with obtaining ethical approval from the Faculty of Nursing and the Ministry of Education; the study proceeded by contacting the principals of the selected schools. The study’s objectives and importance were explained to the students and teachers, and a suitable time for data collection was arranged. Once the appropriate classes for the target age group were identified, consent forms were distributed to the students, who were asked to take them home for parental signature and return them on the day of data collection and blood sampling. A contract was then established with a medical laboratory to carry out the blood draw on‐site at the school, following strict health and safety protocols. On the day of data collection, the consent forms were verified to ensure proper documentation. The researcher and assistants then helped the students fill out the questionnaires, and the students were given 15–20 min to complete the questionnaires. Afterward, blood samples were drawn in a private room, with careful attention to privacy and sent with specialized lab technicians to the laboratory for analysis. Once the laboratory results were received, each set of the results was matched with the corresponding student’s questionnaire to ensure accurate data linkage. It is important to note that the results were provided to the students after coordinating with the school administration, ensuring that each student was informed of their lab results.

The data collection phase lasted for a total of 4 weeks, with 1 week allocated to each school within each directorate. It commenced on Sunday, 1 February 2024, and concluded on 1 March 2024.

### 2.7. Ethical Approval

The researcher had requested approval from the Ethics Committee of Mutah University’s Faculty of Nursing and obtained permissions from the Ministry of Education and the principals of the selected schools to carry out the study in accordance with international ethical research guidelines. Subsequently, after being fully informed about the study’s objectives and benefits, the students and their parents provided their informed written consent. Confidentiality and voluntary participation were emphasized when explaining the study’s purpose to each participant. The confidentiality and anonymity of the information were assured and maintained. Ultimately, the data were securely stored, with access restricted to the research team.

### 2.8. Data Analysis

Using IBM SPSS version 26, the data were analyzed, cleaned, and coded. Frequencies, proportions, and standard deviations (SDs) were calculated, with a significance level set at a *p* value of < 0.05. Descriptive statistics were specifically computed for the mean, SD, and proportion (%) of numeric values. The data were evaluated for relationships between the study variables through cross‐tabulation and chi‐square tests with significance set at the 0.05 level. Hb and ferritin measurements served as the primary outcome variables.

## 3. Results

The Results section is organized into two main parts: sociodemographic characteristics and health history, and factors associated with anemia.

### 3.1. Part 1: Sociodemographic Characteristics and Health History

As shown in Table 1, this study included 284 girls, aged 13–17 years, with an average age of 15.4 years (±1.27). Regarding family income, over half (53.4%) reported that their monthly income was between 200 and 500 Jordanian Dinars. The majority of the parents of the participants—77.8% of fathers and 59.2% of mothers—had completed secondary school. In terms of work, the majority of mothers were unemployed (77.4%), whereas a significant percentage of fathers was employed (83%).

**Table 1:** Demographic characteristics of participants.

Characteristic	*n* available	Category	No.	%
Age (years)	284	13–15	122	43.0
		16–17	162	57.0
Family income (JD^∗^)	206	≤ 200	50	24.3
		200–500	110	53.4
		500–1000	37	18.0
		> 1000	9	4.4
Father’s education	270	Illiterate	18	6.7
		Secondary	210	77.8
		Bachelor and higher	42	15.6
Mother’s education	267	Illiterate	50	18.7
		Secondary	158	59.2
		Bachelor and higher	59	22.1
Father’s occupation	264	Employed	219	83.0
		Unemployed	45	17.0
Mother’s occupation	266	Employed	60	22.6
		Unemployed	206	77.4
Family size	276	< 5	39	14.1
		> 5	237	85.9
Residence	281	Urban	92	32.7
		Rural	189	67.3
BMI category	254	Underweight	90	35.4
		Normal weight	128	50.4
		Overweight	25	9.8
		Obese	11	4.4

Abbreviation: JD^∗^, Jordan Dinar.

Table 2 shows that over half of the students were unsure whether they had IDA. The majority reported not having any chronic conditions, with only 4.9% indicating they had blood disorders, 3.5% reporting intestinal parasites or worms, and 39.6% having a family history of diseases.

**Table 2:** Health histories of participants and their families.

History	No.	%
**IDA** Yes No I don’t know	23 102 158	8.2 36 55.8
**Chronic diseases** Yes No I don’t know	5 236 42	1.8 83.4 14.8
**Blood diseases** Yes No I don’t know	14 151 118	4.9 53.4 41.7
**Intestinal parasites and worms** Yes No I don’t know	10 208 65	3.5 73.5 23
**Family diseases** Yes No	109 166	39.6 60.4

Table 3 indicates that the participants always reported experiencing the following signs and symptoms: 31.8% reported dizziness, 21.8% reported paleness, 45.1% reported headaches, 51.7% reported fatigue, 52.7% reported tachypnea, 54% reported hair loss, 54.2% reported difficulty concentrating, and 36.1% reported coldness in their extremities.

**Table 3:** Signs and symptoms of IDA among participants.

Symptom	Frequency	No.	%
Dizziness	Always	82	31.8
	Sometimes	176	68.2
Pale	Always	49	21.8
	Sometimes	176	78.2
Headache	Always	115	45.1
	Sometimes	140	54.9
Fatigue	Always	120	51.7
	Sometimes	112	48.3
Tachypnea	Always	127	52.7
	Sometimes	114	47.3
Alopecia	Always	129	54.0
	Sometimes	110	46.0
Loss of concentration	Always	129	54.2
	Sometimes	109	45.8
Coldness of extremities	Always	84	36.1
	Sometimes	149	63.9

According to Table 4, most students had not conducted ferritin and Hb tests previously (69.9% and 86.2%, respectively).

**Table 4:** Anemia tests conducted by participants.

Blood test	No.	%
**Ferritin** Yes No	81 188	30.1 69.9
**Hemoglobin** Yes No	37 232	13.8 86.2
**Other** Yes No	60 210	22.2 77.8

Table 5 shows the menstrual period status among the participants. The majority (68.3%) experienced menarche after the age of 12. In addition, 40.3% reported light blood loss during their period, 69.2% experienced dysmenorrhea, 23.4% had hormonal disturbances, and over two‐thirds reported experiencing depression during their period.

**Table 5:** Menstrual period status of participants.

Status	No.	%
**Menarche age** ≤ 12 12 years ≥ 12	46 23 149	21.1 10.6 68.3
**Blood loss** Menorrhagia Light blood loss I don’t know	79 106 78	30 40.3 29.7
**Dysmenorrhea** Yes No I don’t know	184 66 16	69.2 24.8 6
**Hormonal disturbances** Yes No I don’t know	61 114 86	23.4 43.7 33
**Depression** Yes No I don’t know	174 67 22	66.1 25.5 8.4
**Other S&S** Yes No I don’t know	104 139 20	39.5 52.9 7.6

### 3.2. Part 2: Factors Associated With Anemia

Table 6 indicates that an analysis of dietary habits in relation to Hb levels showed that most dietary variables—including frequency of meals, red meat, fish, fresh fruit, and vegetable consumption—were not significantly associated with anemia status (*p* > 0.05). However, the consumption of grains at least once a week demonstrated a significant association (*p* = 0.001), indicating that regular grain intake may have a positive relationship with normal Hb levels. Overall, while most dietary patterns did not show a statistically significant effect, grain consumption appeared to be an important dietary factor influencing anemia status among participants.

**Table 6:** Relationship between participants’ dietary characteristics and hemoglobin levels.

Dietary	HB			*P* value
	Moderate	Mild	Normal	
**3 meals/day** Yes No	2 (0.7%) 14 (5.1%)	13 (4.7%) 44 (16.0%)	57 (20.7%) 138 (50.2%)	0.755
**Red meat** Yes No Only chicken	5 (1.8%) 7 (2.5%) 5 (1.8%)	4 (1.4%) 20 (7.2%) 35 (12.6%)	36 (13.0%) 72 (26.0%) 92 (33.2%)	0.173
**Fish** Yes No	4 (1.5%) 13 (4.7%)	15 (5.5%) 42 (15.3%)	72 (26.2%) 127 (46.2%)	0.436
**Fresh fruit** Yes No	5 (1.9%) 12 (4.4%)	24 (8.9%) 34 (12.6%)	99 (36.7%) 96 (35.6%)	0.116
**Green vegetable** Yes No	0 (0.0%) 17 (6.8%)	8 (3.2%) 47 (18.7%)	37 (14.7%) 142 (56.6%)	0.214
**Vegetables once/week** Yes No	14 (5.2%) 3 (1.1%)	52 (19.3%) 6 (2.2%)	176 (65.4%) 18 (6.7%)	0.543
**Fruits once/week** Yes No	13 (4.8%) 4 (1.5%)	42 (15.6%) 16 (5.9%)	161 (59.6%) 34 (12.6%)	0.221
**Grain/once week** Yes No	6 (2.3%) 11 (4.1%)	24 (9.0%) 34 (12.8%)	124 (46.6%) 67 (25.2%)	0.001
**Milk/once week** Yes No	9 (3.4%) 8 (3.0%)	37 (13.9%) 21 (7.9%)	125 (46.8%) 67 (25.1%)	0.605

Table 7 presents the results of anemia diagnostic tests by age group. The data reveal that the overall prevalence of IDA was 21.5%, with 6.3% of cases classified as moderate anemia and 15.2% as mild anemia. A statistically significant difference was observed in Hb levels between the age groups (*p∼0.001*), indicating that older adolescents (16–17 years) had better Hb status compared to younger adolescents (13–15 years). However, no significant differences were found in ferritin (*p* = 0.125) or MCV (*p* = 0.274) between the age groups, suggesting that while Hb levels improve with age, ferritin and MCV remain relatively consistent across both groups.

**Table 7:** Anemia diagnostic tests by age group.

Test	Category	13–15 Years	%	16–17 Years	%	Total (*n* = 284)	%	*p* value
Hemoglobin (Hb)	Moderate anemia	2	0.7	15	5.3	17	6.0	*∼0.001*
	Mild anemia	14	4.9	45	15.8	59	20.8	
	Normal	106	37.3	102	35.9	208	73.2	
Ferritin (ng/mL)	< 12	37	13.1	63	22.3	100	35.3	0.125
	12–150	85	30.0	98	34.6	183	64.7	
MCV (fL)	< 80	30	10.6	50	17.6	80	28.2	0.274
	80–100	91	32.0	112	39.4	203	71.5	
	> 100	1	0.4	0	0.0	1	0.4	

The results of Table 8 indicate a significant association between family income and Hb levels (*p* = 0.001), with lower‐income students showing higher rates of moderate and mild anemia. No anemia cases were observed in the highest income group. Ferritin and MCV levels did not show statistically significant differences across income groups (*p* = 0.227 and *p* = 0.363, respectively), though low ferritin was more common in lower‐income students. Overall, lower socioeconomic status appears to be linked to higher anemia prevalence, particularly reflected in Hb levels.

**Table 8:** Anemia diagnostic tests by family income.

Test	< 200 JD		200‐< 500JD		500‐1000 JD		> 1000JD		*P* Value
**HB** Moderate Mild Normal	1 13 36	0.5% 6.3% 17.5%	3 34 73	1.5% 16.5% 35.4%	7 6 24	3.4% 2.9% 11.7%	0 0 9	0.0% 0.0% 4.4%	0.001
**Ferritin** **< 12** **12–150**	25 25	12.2% 12.2%	36 73	17.6% 35.6%	15 22	7.3% 10.7%	3 6	1.5% 2.9%	0.227
**MCV** < 80 80–100	11 39	5.3% 18.9%	29 81	14.1% 39.3%	13 24	6.3% 11.7%	4 5	1.9% 2.4%	0.363

Table 9 indicates that analysis of anemia diagnostic parameters in relation to parental employment status revealed no statistically significant associations. HB and MCV levels did not differ significantly between participants with employed and unemployed parents. However, ferritin levels demonstrated a borderline association with paternal and maternal employment status (*p* = 0.058 and *p* = 0.089, respectively), indicating a possible trend toward lower iron stores among students whose parents were unemployed. Overall, the findings suggest that parental employment status was not a significant determinant of anemia‐related hematological indices in the study population.

**Table 9:** Anemia diagnostic tests by parents’ employment status.

Variable	Category	Father unemployed *n* (%)	Father employed *n* (%)	*p*‐value	Mother unemployed *n* (%)	Mother employed *n* (%)	*p*‐value
Hemoglobin (HB)	Moderate Mild Normal	3 (1.1%) 9 (3.4%) 33 (12.5%)	14 (5.3%) 47 (17.8%) 158 (59.8%)	0.976	13 (4.9%) 40 (15.1%) 153 (57.7%)	4 (1.5%) 18 (6.8%) 37 (14.0%)	0.178
Ferritin (μg/L)	< 12 12–150	22 (8.4%) 23 (8.7%)	74 (28.1%) 144 (54.8%)	0.058	69 (26.1%) 136 (51.5%)	27 (10.2%) 32 (12.1%)	0.089
MCV (fL)	< 80 80–100 > 100	13 (4.9%) 32 (12.1%) 0 (0.0%)	63 (23.9%) 155 (58.7%) 1 (0.4%)	0.902	54 (20.4%) 151 (57.0%) 1 (0.4%)	23 (8.7%) 36 (13.6%) 0 (0.0%)	0.147

## 4. Discussion

Anemia is a significant public health issue for adolescent girls in developing countries, affecting their growth, pregnancy outcomes, and long‐term health [19]. IDA is the leading cause, accounting for more than 50% of anemia cases. Women of reproductive age are particularly prone to iron deficiency because of the increased iron needs during pregnancy, lactation, menstrual blood loss, and insufficient nutrition throughout their reproductive years [20]. This study aimed to determine the prevalence of IDA among adolescent schoolgirls in Southern Jordan, as well as to identify the factors associated with anemia. The study findings indicated that the prevalence of IDA among adolescent girls in Jordan was found to be 21.5%, encompassing both mild and moderate cases. These results align with a study conducted in Jatinangor, Indonesia, which examined IDA and its associated factors among adolescent girls and women in a rural area. In that study, the prevalence of IDA among the girls was reported to be 21.1% [2]. This finding was also comparable to two studies conducted in the Dembia District and Sudan, which reported prevalence rates of 25.5% and 24.3%, respectively [21, 22].

On the other hand, this prevalence is lower than the global average. According to the World Health Organization, in 2019, the global prevalence of anemia in women aged 15–49 was 29.9% (95% uncertainty interval: 27.0%–32.8%), equating to over half a billion women affected [23]. This rate is also lower than that reported in an eastern Africa study that examined the prevalence and contributing factors of anemia in women of reproductive age, where the prevalence was 34.85% [20]. In addition, a study conducted in Lahore aimed to assess the magnitude and status of anemia in adolescent girls with a high prevalence of anemia. Severity was assessed through concentration of Hb in blood, revealing that 43% of participants had severe anemia, with the majority (36%) belonging to the low‐ and middle‐income class [24].The observed disparity in the prevalence of IDA may be attributed to differences in socioeconomic status across South Africa, Lahore, and Jordan, as these factors can significantly influence health outcomes. In addition, variations in dietary practices could also contribute to these differences. It is also important to highlight that the prevalence rate of IDA in this study was lower compared to a previous study conducted in Nepal. The study in Nepal, which aimed to determine the prevalence of anemia and its associated factors among school‐going adolescent girls in Dhankuta municipality, found a prevalence rate of 18% [25].

Variations in anemia rates may arise from differences in nutrition, healthcare availability, education, and economic status. Individuals in lower socioeconomic conditions often face higher anemia rates because of restricted access to nutrient‐rich foods and adequate healthcare. Furthermore, prevalence rates can differ by region, influenced by factors such as public health standards, dietary quality, cultural traditions, eating habits, and the effectiveness of local health programs.

Our study also found that the highest prevalence of both moderate and mild anemia was observed in 16‐year‐old girls. This finding contrasts with a study conducted in rural secondary schools in Osmanabad district, Maharashtra, which estimated the prevalence of anemia among school‐going adolescent girls aged 11–16 years. In that study, the majority of anemic girls were 11 years old, followed by 16‐year‐olds, with the lowest prevalence found in 12‐year‐old girls [26]. Older adolescents (16–17 years) showed a higher prevalence of mild and moderate anemia compared to younger students (13–15 years), likely because of increased iron requirements during growth and, for girls, menstrual blood loss. Although ferritin and MCV did not differ significantly, Hb levels suggest that older adolescents are at greater risk of anemia if dietary iron is inadequate. These findings highlight the need for targeted interventions, including school‐based nutrition programs, iron supplementation, and education on iron‐rich diets and absorption‐enhancing practices, to prevent anemia and improve overall health and quality of life in this age group.

Ensuring adequate nutrition in adolescence is essential for long‐term well‐being, as it helps disrupt the cycle of malnutrition, chronic diseases, and poverty [27]. The nutritional assessment in this study revealed that most dietary factors—including meal frequency and the intake of red meat, fish, fruits, and vegetables—were not significantly associated with anemia status (*p* > 0.05). These findings contrast with those of a study conducted among adolescent girls at Project High School in Bahadurganj, Bihar [28], which reported a significant association between dietary patterns and the prevalence of anemia. In that study, anemia was more common among participants who rarely consumed iron‐rich foods such as red meat, poultry, fish, and fortified cereals. In spite of the suboptimal dietary habits observed, the prevalence of anemia in this study was considered moderate, which is lower than what is typically expected in similar populations. These findings suggest that, although the participants’ diet was not optimal, their overall nutritional intake may have been sufficient to prevent more severe forms of anemia. Findings reveal a significant relationship between family income and Hb levels (*p* = 0.001). Students from lower‐income families showed a higher occurrence of moderate and mild anemia, while no anemia cases were observed among those in the highest income group. This finding aligns with a study on factors associated with anemia among adolescent girls in Kampung Sawah, South Tangerang [29], which reported significant associations between anemia and several variables, including family income, knowledge, intake of foods that promote iron absorption, nutritional status, and physical activity.

### 4.1. Implications

This study’s findings have important implications for both nursing education and practice, particularly in the fields of adolescent health, nutrition, and preventive care. It underscores the high prevalence of IDA among adolescent girls, highlighting the critical need for nurses to be adequately trained to recognize, assess, and manage anemia in this at‐risk group. In terms of nursing education, the study could influence curriculum development by emphasizing the significance of early screenings, nutrition‐focused education, and community‐based strategies. For nursing practice, it stresses the need for nurses to advocate for school health programs, collaborate with interdisciplinary teams, and offer culturally relevant education to encourage healthy eating habits and prevent iron deficiency. Furthermore, the study suggests that nurses should take an active role in raising awareness of anemia and supporting routine health screenings in schools for early detection and effective management.

## 5. Recommendations

1. Nutrition‐Focused Interventions
  ◦ Implement school‐based programs that provide iron‐rich meals or snacks.
  ◦ Promote the consumption of iron‐rich foods, such as meat, legumes, and leafy vegetables, and educate students on combining them with vitamin C–rich foods to enhance absorption.
2. Health Education and Awareness
  ◦ Conduct workshops for students, parents, and teachers on anemia prevention and the importance of balanced diets.
  ◦ Educate adolescents on dietary habits that may inhibit iron absorption, such as drinking tea immediately after meals.
3. Screening and Supplementation
  ◦ Conduct regular Hb and ferritin screenings for adolescents, with particular attention to older girls (16–17 years).
  ◦ Provide iron supplementation programs for at‐risk students in accordance with recommendations from local health authorities.
4. Policy Recommendations
  ◦ Encourage educational and health authorities to integrate nutrition and anemia prevention programs into school curricula.
  ◦ Support policies that improve access to affordable, iron‐rich foods for families from lower socioeconomic groups.

These *context-specific recommendations* aim to reduce the prevalence of anemia, enhance adolescent health, and support sustainable improvements in nutritional outcomes.

### 5.1. Limitations

This study has several limitations that should be considered when interpreting the results. First, the cross‐sectional design restricts the ability to draw causal conclusions between the identified factors and the occurrence of IDA. Second, as the data were collected from a specific region (Southern Jordan), the findings may not be generalizable to other areas or countries with different socioeconomic, cultural, or dietary contexts. Third, the use of self‐reported questionnaires for dietary habits introduces the potential for recall bias, as participants may inaccurately report their food intake. In addition, although blood tests were used to diagnose anemia, they may not fully account for other influences on iron status, such as underlying health conditions, infections, or chronic illnesses. The study also did not evaluate the long‐term effects or sustainability of school‐based health interventions, which would provide further insight into the lasting impact of such programs. Finally, excluding female students with heavy or clotted menstrual cycles to avoid confounding effects on Hb and iron levels may have slightly underestimated the overall prevalence of anemia.

## 6. Conclusions

IDA affects 21.5% of adolescent girls in Southern Jordan, with older adolescents (16–17 years) being most vulnerable. Age‐targeted, school‐based programs that provide nutritional education, promote iron‐rich diets, and ensure regular anemia screening are essential. Combined with policies improving access to nutritious foods, these interventions can help prevent IDA and improve the health and well‐being of adolescent girls in the region.

## Conflicts of Interest

The author declares no conflicts of interest.

## Funding

The researcher also thanks Mutah University for their financial support in facilitating both the data collection process and the laboratory blood sampling work.

## Data Availability

The data that support the findings of this study are available on request from the corresponding author. The data are not publicly available because of privacy or ethical restrictions.
